# Evaluation of textile substrates for dispensing synthetic attractants for malaria mosquitoes

**DOI:** 10.1186/1756-3305-7-376

**Published:** 2014-08-16

**Authors:** Collins K Mweresa, Wolfgang R Mukabana, Philemon Omusula, Bruno Otieno, Tom Gheysens, Willem Takken, Joop JA van Loon

**Affiliations:** International Centre of Insect Physiology and Ecology, P.O. Box 30772–00100, Nairobi, Kenya; Laboratory of Entomology, Wageningen University and Research Centre, P.O. Box 8031, Wageningen, EH 6700 The Netherlands; School of Biological Sciences, University of Nairobi, P.O. Box 30197–00100, Nairobi, GPO, Kenya; Department of Textiles, University of Gent, Technologiepark-Zwijnaarde 907, Gent, Belgium

**Keywords:** *Anopheles gambiae*, *Anopheles funestus*, IB1-impregnated nylon, Polyester, Cotton, Cellulose, Sodium polyacrylate, Attraction, Trapping, Kenya

## Abstract

**Background:**

The full-scale impact of odour-baited technology on the surveillance, sampling and control of vectors of infectious diseases is partly limited by the lack of methods for the efficient and sustainable dispensing of attractants. In this study we investigated whether locally-available and commonly used textiles are efficient substrates for the release of synthetic odorant blends attracting malaria mosquitoes.

**Methods:**

The relative efficacy of (a) polyester, (b) cotton, (c) cellulose + polyacrylate, and (d) nylon textiles as substrates for dispensing a synthetic odour blend (Ifakara blend 1(IB1)) that attracts malaria mosquitoes was evaluated in western Kenya. The study was conducted through completely randomized Latin square experimental designs under semi-field and field conditions.

**Results:**

Traps charged with IB1-impregnated polyester, cotton and cellulose + polyacrylate materials caught significantly more female *Anopheles gambiae sensu stricto* (semi-field conditions) and *An. gambiae* sensu lato (field conditions) mosquitoes than IB1-treated nylon (P = 0.001). The IB1-impregnated cellulose + polyacrylate material was the most attractive to female *An. funestus* mosquitoes compared to all other dispensing textile substrates (P < 0.001). The responses of female *An. funestus* mosquitoes to IB1-treated cotton and polyester were equal (P = 0.45). Significantly more female *Culex* mosquitoes were attracted to IB1-treated cotton than to the other treatments (P < 0.001). Whereas IB1-impregnated cotton and cellulose + polyacrylate material attracted equal numbers of female *Mansonia* mosquitoes (P = 0.44), the catches due to these two substrates were significantly higher than those associated with the other substrates (P < 0.001).

**Conclusion:**

The number and species of mosquitoes attracted to a synthetic odour blend is influenced by the type of odour-dispensing material used. Thus, surveillance and intervention programmes for malaria and other mosquito vectors using attractive odour baits should select an odour-release material that optimizes the odour blend.

## Background

The use of semiochemicals as a novel means of monitoring and controlling mosquito vectors has been investigated under different environmental conditions with promising results [1–4]. This technology is pegged on the understanding that blood-questing mosquitoes are mainly guided to their hosts by olfactory cues [5, 6]. Indeed, host-specific attractant compounds have been identified and constituted into synthetic odour blends to provide a complementary tool for sampling and control of both outdoor- and indoor-biting malaria mosquitoes [7–10]. However, improvement of odour-baited trapping systems depends partly on efficacy and sustainability of selected odour-dispensing devices [11, 12]. Importantly, devices used to dispense odorants should ensure stability of impregnated active ingredients, sustained release of optimal odour concentrations and be easy to prepare for large-scale application [11, 13].

Recent findings have shown that nylon strips treated with synthetic attractant odorants lured significantly higher numbers of host-seeking *Anopheles gambiae* Giles *sensu stricto* (hereafter referred to as *An. gambiae*) mosquitoes into traps than glass vials and low density polyethylene (LDPE) sachets containing the same attractants [13, 14]. Like other repellent- or insecticide-impregnated fabric materials [15–17], nylon strips impregnated with attractant odorants have also demonstrated a long-term residual activity to *An. gambiae* for over one year post-treatment under semi-field conditions [18]. Besides nylon, cotton socks have been utilised to collect human foot odour in experiments evaluating the attraction of *An. gambiae* [19–21]. In addition, suitability of both polyester and cotton materials to dispense a candidate contaminant insecticide inside an odour-baited station against wild malaria mosquitoes in southern Tanzania was demonstrated [22]. The absorption layer of commonly used unscented, ultra-thin disposable sanitary pads consists of cellulose + polyacrylate for holding absorbed liquids. These materials have high capacity to absorb fluids, however, it is not known whether such readily available materials would also be effective in dispensing synthetic attractants optimised to lure malaria vectors into trapping tools. To answer this question, we investigated whether locally available and commonly used polyester netting, cotton clothing and cellulose + polyacrylate materials provided similar or better release matrices for synthetic attractants to host-seeking mosquitoes compared to nylon.

## Methods

### Mosquitoes

The Mbita strain of female *An. gambiae* mosquitoes was used for semi-field experiments conducted between November 2011 and April 2012 within a screen-walled greenhouse measuring 11.4 m × 7.1 m × 2.8 m, with the roof apex standing at 3.4 m high. The mosquitoes were reared in the insectary at the Thomas Odhiambo Campus (TOC) of the International Centre of Insect Physiology and Ecology (*icipe*) located at Mbita Point, western Kenya. Adult mosquitoes were kept in 30 cm^3^ gauze cages, fed on a human arm for a blood meal and provided with 6% glucose solution supplied through a Whatman filter paper wick. Female mosquitoes oviposited on a wet filter paper placed in a Petri dish. The eggs were thereafter dispensed in plastic trays half-filled with water obtained from Lake Victoria. Larvae were fed on Tetramin® baby fish food provided thrice a day. Pupae were collected daily and transferred into 30 cm^3^ gauze cages for emergence. A total of 200 adult female mosquitoes aged 3–5 d old without prior access to a blood meal were randomly aspirated and kept in a plastic holding cup for each experiment (20:00–06:30 h). The mosquitoes were starved for 8 h while being supplied with water through a wet cotton towel placed on top of the cage before they were released at the centre of a screen-walled greenhouse. The roof of the greenhouse was covered with a glass panel whereas a large mosquito netting cage was suspended inside from the roof along the screened wall to a sand-covered floor [21].

### Field study site

Field studies were carried out at Kigoche village (00°34’S, 034°65’ E and 1158 m above sea level) in May-June 2012. The village is situated near Ahero town, in the Kano flood plains of Kisumu County, western Kenya, approximately 110 km east of the *icipe* -TOC where all semi-field experiments were conducted. Annual rainfall ranges from 1000–1800 mm, temperatures between 17 - 32°C and 65% average relative humidity (RH) are experienced. The long rainy season occurs between March and August while short rains are common in October-November. Ahero is a seasonally inundated flood plain adjacent to the River Nyando within the Lake Victoria basin in western Kenya. Irrigated rice farming is the dominant economic activity, but traditional farming of maize, millet, bananas, sweet potatoes, beans, cassava, sorghum and rearing of indigenous cattle, goats, sheep and poultry is also practiced. Malaria is transmitted primarily by *An. funestus* Giles*, An. gambiae s.s.* and *An. arabiensis* Patton [10, 23].

### Description of study houses

A total of five houses, each measuring between 15.8 and 22.5 m^2^ in ground surface area, were selected by using computer-generated random numbers and labelled for trapping of outdoor mosquito populations. The houses consisted of mud walls and floors with open eaves, corrugated iron-sheet roofs, no ceiling, and they were either single or double roomed [24]. They were located on a transect oriented east–west along the northern edge of the Ahero rice irrigation scheme, approximately 28–150 m apart, 10–20 m away from cowsheds and within a range of 100 m from irrigation water channels and rice paddies [8, 10]. The exact location of all houses was determined with a hand-held global positioning system receiver (Trex HC series, Garmin International, USA). The prevailing outdoor temperature, RH and rainfall were recorded from a weather station located at the Ahero Irrigation Research Station (AIRS), located approximately 800 m away from the study houses. During experimental nights, the five houses were occupied routinely by 2–5 dwellers who slept under bed nets without insecticides or repellents [25].

### Preparation and dispensing of synthetic mosquito lures

A synthetic mosquito attractant blend called Ifakara blend 1 (IB1) was made from 10 chemicals [8, 14] and supplemented with carbon dioxide. The carbon dioxide was produced nightly from a mixture of 2 L of tap or river water, 17.5 g of instant dry yeast [10, 26] and 250 cm^3^ of molasses (44.7% pure, containing 34.2% sugar and 76.4% of total dissolved solids). Molasses is a by-product formed after crystallization of refined white sugar from raw sugarcane syrup (Mumias Sugar Company Ltd, Kenya).

Nylon strips have been used to dispense synthetic attractant odorants for studies on host-seeking mosquitoes [13, 14]. Since the absorption layer embedded within a disposable sanitary pad was 24 cm long, this length was adopted for all four types of release substrates evaluated in the present study. A total of ten individual strips (1 × 24 cm) were cut from (a) nylon stockings (15 denier microfibre, 90% polyamide and 10% spandex purchased from Bata Shoe Company Ltd, Kenya), (b) 100% polyester mosquito bed-net without insecticide (Country Mattresses Company Ltd, Kenya), (c) 100% woven cotton (Articot Golden quality duster, India) and (d) the absorbent layer (95% cellulose and 5% sodium polyacrylate fibres) of a disposable menstrual sanitary pad (unscented *Always* ultra thin, ultra-fine Gel-X, Fabricadona Egiptopor, EG Procter & Gamble Company, Egypt). Currently, sodium polyacrylate is the cheapest and most commonly used super absorbent polymer on the market. The composition of the absorbent layer embedded within the sanitary pad was determined at the Department of Textiles at Ghent University, Belgium.

Each of the ten strips from the four substrates was separately soaked in a glass bottle containing 1 ml of an optimal concentration of the individual chemical constituents of blend IB1 [8, 14]. Thereafter, the strips were air-dried at room temperature for 5 h. All attractant-treated strips for each of the four substrates were hooked at one end and hung inside the odour plume tubes of separate Mosquito Magnet-X (MM-X) counter flow geometry traps (American Biophysics, North Kingstown, RI, USA). Traps containing IB1 dispensed from any of the four substrates were supplied with carbon dioxide (approximately 81 ml/min) through 5 mm-wide silicon tubing during each experimental night. However, 10 untreated strips (no odour bait) as control were cut from each substrate soaked in 1 ml of water, air-dried for 5 h and tested during preliminary investigations against attractant-impregnated substrates.

Each trap was suspended on a separate tripod stand within a screen-walled greenhouse or under the eaves of a village house with its trap opening positioned 15 cm above ground level, marked and used for one specific treatment throughout the experiment [25, 27, 28]. The traps were operated on 12 V and sequentially alternated between or among houses on a nightly basis, thereby reducing potential bias due to house location or house characteristics. Individual sets of attractant-impregnated substrates were separately stored at 4°C between experimental runs. Latex examination gloves were worn when cutting and impregnating strips, and also when hanging them inside the plume tube of specified traps to avoid contamination from human volatiles. Prevailing temperature and RH levels in the greenhouse were recorded at an interval of 30 min using a data logger (Tinytag® Ultra, model TGU-1500, INTAB Benelux, The Netherlands).

### Responses of *An. gambiae*to untreated and attractant-treated substrates under semi-field conditions

Although nylon has been confirmed to be a more effective matrix for dispensing synthetic mosquito attractants than LDPE sachets, we performed preliminary experiments to investigate whether alternative locally available materials performed similarly or better [14, 22]. Treatments used in the first sets of competitive dual-choice assays included (a) nylon versus IB1-treated nylon, (b) polyester versus IB1-treated polyester, (c) cotton versus IB1-treated cotton, and (d) cellulose + polyacrylate versus IB1-treated cellulose + polyacrylate material. Additional dual-choice assays were conducted to compare behavioural responses of *An. gambiae* to blend IB1 dispensed from nylon versus blend IB1 released from polyester, cotton and cellulose + polyacrylate material. Individual bioassays were run for four nights and the traps were diagonally placed within the screen-house at a distance of 13.0 m apart. Each untreated (control) and IB1-treated substrate was re-used throughout the four experimental nights [14].

### Responses of *An. gambiae*to attractant-treated substrates under semi-field conditions

The efficacy of different substrates to dispense chemical constituents of blend IB1 for attraction of *An. gambiae* was tested further in a semi-field enclosure through a completely randomized 4 × 4 Latin square experimental design replicated over 16 consecutive nights. The design included blend IB1 dispensed from (a) nylon as a positive control, (b) polyester, (c) cotton, and (d) cellulose + polyacrylate material. The traps were placed at a distance of 5.0 m or 9.2 m apart. After this experiment, all IB1-treated substrates were subsequently deployed for luring outdoor-biting malaria and other mosquitoes into traps for 25 nights at Kigoche village.

### Efficacy of attractant-treated substrates to lure malaria and other mosquitoes in the field

The potential of traps containing IB1-treated substrates to intercept and attract outdoor mosquitoes under eaves of village houses occupied by the dwellers overnight was tested in a 5 × 5 Latin square experimental assay for 25 successive nights (18:30–06:30 h). The treatments included (a) an unbaited MM-X trap (no odour bait), (b) IB1-treated nylon, (c) IB1-treated polyester, (d) IB1-treated cotton, and (d) IB1-treated cellulose + polyacrylate material. The attractant-impregnated substrates were re-used for the entire study period of 25 nights and had previously been tested under semi-field conditions for 16 nights post-impregnation. The houses selected for trapping of mosquitoes outdoors were spaced within a distance range of 28 – 150 m apart. Variations due to house characteristics were reduced by ensuring that the treatments were equally rotated among the five houses daily.

At the end of each experimental night, all traps were transported to a field laboratory located at the Ahero Multipurpose Development Training Institute (AMDTI) (approximately 5 km away) and placed in a freezer for 30 min. The frozen adult mosquitoes were emptied into labeled Petridishes, identified morphologically [29], counted, and recorded according to (i) sex i.e. male or female *An. gambiae* s.l. *An. funestus*, *Culex, Mansonia* spp. and other anopheline mosquitoes (all collected *Anopheles* species except *An. gambiae* s.l. and *An. funestus*) and (ii) external abdominal appearance as unfed, blood-fed or gravid female *An. gambiae* s.l. and *An. funestus* [30]. All female *An. gambiae* s.l. and *An. funestus* were separately preserved in 2 ml Eppendorf tubes containing silica gel crystals and labelled. A randomly selected sub-sample of 125 females of *An. gambiae* s.l*.* from all treatments was analysed for species composition using a ribosomal Polymerase Chain Reaction (PCR) assay [31].

### Ethical approval

Scientific and ethical clearance of the study was granted by the Kenya Medical Research Institute (KEMRI/RES/7/3/1). Inclusion consent of houses into the study was obtained from household heads and the local (village-level) administration.

### Data analysis

The response variable was the number of mosquitoes trapped. Differences between proportions of *An. gambiae* caught in both traps during dual-choice bioassays were analysed using a Chi-square test to determine whether the proportion of mosquitoes caught in each of the two MM-X traps differed from a 1:1 distribution. A generalized Linear Model fitted with a Poisson regression and a logarithmic link function was used to investigate the effect of treatment on behavioural responses of mosquitoes to blend IB1 dispensed from different substrates and tested in the 4 × 4 or 5 × 5 Latin square experimental bioassays [9]. Effects were considered to be significant at P < 0.05. The effects of treatment and house location on mosquito catches were tested as parameters in the model. Day was fitted as a random factor in the mixed effects GLM. All analyses were carried using IBM SPSS statistical software, version 16.

## Results

### Responses of *An. gambiae*to untreated and IB1-treated substrates under semi-field conditions

**Figure 1:**
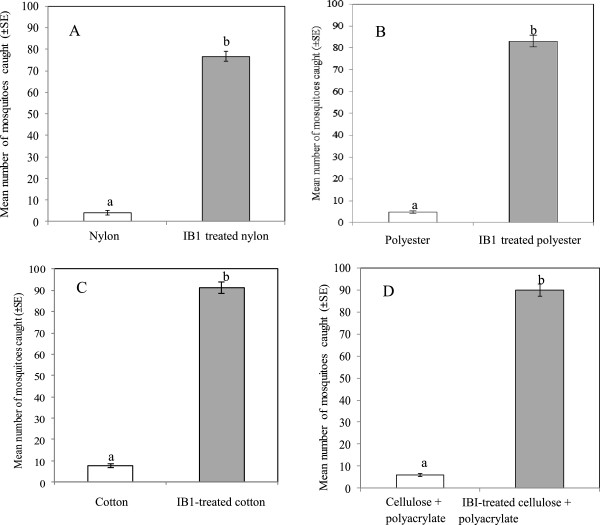
**Mean number ± SE of female** ***An. gambie*** **caught in a dual-choice assay between untreated nylon and IB1-treated nylon (panel A), untreated polyester and IB1-treated polyester (panel B), untreated cotton and IB1-treated cotton (panel C) and untreated cellulose + polyacrylate and IB1-treated cellulose + polyacrylate embedded within the sanitary pad (panel D) material for four nights.** Mean mosquito catches with different letters in the same panel differ significantly (P < 0.05).

Semi-field experiments were conducted between November 2011 and April 2012 at an average temperature and RH of 25.7 ± 2.5°C and 62.8 ± 8.4%, respectively. The attractiveness of nylon, polyester, cotton, and cellulose + polyacrylate material to *An. gambiae* was significantly enhanced by treatment with blend IB1 (P < 0.001). The total mosquito catches with untreated and treated textile substrate materials were as follows: (a) untreated nylon (n = 18, 6%) and IB1-treated nylon (n = 284, 94%), (b) untreated polyester (n = 20, 6%) and IBI-treated polyester (n = 325, 94%), (c) untreated cotton (n = 31, 8%) and IB1-treated cotton (n = 362, 92%), and (d) untreated cellulose + polyacrylate (n = 24, 6%) and IB1-treated cellulose + polyacrylate material (n = 354, 94%) (Figure 1). A second series of dual-choice bioassays indicated that the responses of *An. gambiae* to IB1-treated nylon were significantly lower compared to IB1-treated polyester (P = 0.001), IB1-treated cotton (P = 0.001) and IB1-treated cellulose + polyacrylate material (P = 0.010) (Table 1).

**Table 1:** **Total and mean ± SE number of female** ***An. gambiae*** **attracted in a dual-choice bioassay by blend IB1 dispensed from nylon (reference treatment) versus candidate odour-dispensing substrates (polyester, cotton and cellulose + polyacrylate material) within a screen-walled greenhouse**

Candidate substrate	N	n	Mean ± SE mosquitoes caught		
			Nylon	Candidate substrate	P-value
Polyester	4	474	42.8 ± 3.3	75.8 ± 4.4	0.001
Cotton	4	434	43.0 ± 3.3	65.5 ± 4.5	0.001
Cellulose + polyacrylate	4	359	35.8 ± 3.0	54.0 ± 3.7	0.010

N is the number of experimental nights, n is the total number of mosquitoes caught and SE is the standard error of the mean catch per night.

### Responses of *An. gambiae*to attractant-treated substrates under semi-field conditions

Of the 3,200 mosquitoes released, 65.2% (n = 2,087) were trapped (Table 2). The catches of *An. gambiae* were influenced by house location (P = 0.001) and type of odour-dispensing substrate (P = 0.001). The responses of mosquitoes to blend IB1 dispensed from nylon were significantly lower compared to cotton (P = 0.014) and cellulose + polyacrylate material (P = 0.001). However, IB1-impregnated nylon was more attractive to mosquitoes than similarly treated polyester but the difference was not statistically significant (P = 0.07). The same treatments were tested in the field for 25 successive nights.

**Table 2:** **Total and mean (±SE) number of female** ***An. gambiae*** **collected in MM-X traps baited with blend IB1 dispensed from nylon, polyester, cotton and cellulose + polyacrylate material within a screen-walled greenhouse**

Treatment	N	Mosquitoes caught	
		n	Mean (±SE)
IB1-treated nylon	16	428	26.8 ± 1.3^a^
IB1-treated polyester	16	377	23.6 ± 1.2^a^
IB1-treated cotton	16	503	31.4 ± 1.4^b^
IB1-treated cellulose + polyacrylate	16	779	48.7 ± 1.7^c^

N is the number of experimental nights, n is the total number of mosquitoes caught whereas SE is the standard error of the mean catch per night. Mean ± SE mosquito catches within the same column assigned different letter superscripts are significantly different at P < 0.05 (Generalized Linear Models).

### Efficacy of attractant-treated substrates to lure malaria and other mosquitoes in the field

#### Female mosquitoes

**Figure 2:**
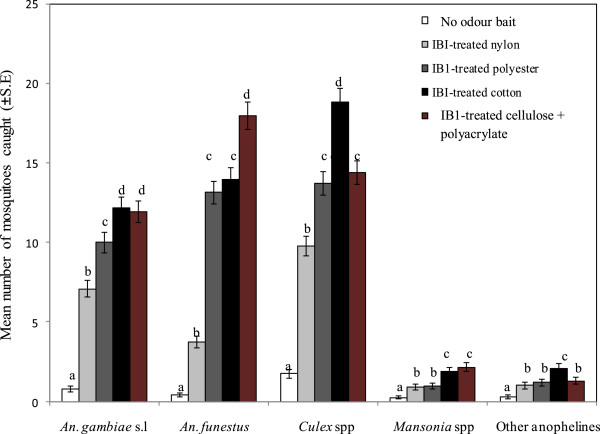
**Mean number ± SE of female mosquitoes caught in an outdoor trap without odour (white square), baited with blend IB1 dispensed from nylon (gray square), polyester (dark gray square), cotton (black square) or cellulose + polyacrylate (brown square) material for 25 nights in Kigoche village.** Mean catches with different letters within the same mosquito group differ significantly (P < 0.05).

An average outdoor temperature of 23.6 ± 3.0°C, 64.4 ± 13.7% RH and a total of 77.6 mm of rainfall (for 18 days) were recorded during the 25 nights of field experiments (May-June 2012). A total of 4,415 mosquitoes were collected outdoors in all traps combined, with 93.6% (n = 4,134) females and 6.4% (n = 281) males. Female mosquitoes comprised *An. gambiae* s.l. *(*25.4%), *An. funestus* (30.2%), *Culex* spp. (36.7%), *Mansonia* spp. (3.9%) and other anopheline spp. (3.9%) (Figure 2).

Trap collections of female *An. gambiae* s.l. were influenced by house location (P = 0.001) and treatment (P = 0.001). The IB1-treated nylon was significantly less attractive to *An. gambiae* s.l*.* than similarly-treated polyester (P = 0.001), cotton (P = 0.001) and cellulose + polyacrylate material (P = 0.001). Although IB1-treated cotton and cellulose + polyacrylate material were the most attractive to *An. gambiae* s.l., catches between both substrates were not different (P = 0.546). Moreover, the cellulose + polyacrylate material was the most effective substrate for dispensing IB1 to *An. funestus* compared to other materials (P < 0.001), whereas nylon was the least effective (P < 0.001) (Figure 2).

There was no difference in the mean numbers of *An. funestus* collected in traps containing IB1 dispensed from cotton and polyester material (P = 0.45). The IB1-treated nylon was significantly less attractive to *Culex* spp. than similarly-treated polyester (P = 0.001), cotton (P = 0.001) and cellulose + polyacrylate material (P = 0.001). Although IB1-treated cotton was the most attractive to *Culex* spp. compared to other materials (P < 0.001), trap collections were not different between IB1-impregnated polyester and cellulose + polyacrylate material (P = 0.53). The attractiveness of blend IB1 dispensed from nylon to *Mansonia* spp. was not different from polyester (P = 0.89), but it was significantly lower than to similarly-treated cotton (P = 0.02) and cellulose + polyacrylate material (P = 0.010). Furthermore, the responses of other anopheline mosquitoes to IB1 dispensed from nylon were not different from polyester (P = 0.52) and cellulose + polyacrylate material (P = 0.72), instead they were lower compared to IB1-treated cotton (P = 0.023).

### Male mosquitoes

The 281 male mosquitoes caught outdoors comprised *An. gambiae* s.l. (50.9%), *An. funestus* (30.6%), *Culex* spp. (14.2%), *Mansonia* spp. *(*1.4%) and other anopheline spp. (2.9%) (Table 3). Whereas traps baited with IB1-treated nylon collected similar catches of *An. gambiae* s.l. as the control (no odour) (P = 0.87), IB1-treated nylon was significantly less attractive than similarly-treated polyester (P = 0.001), cotton (P = 0.015) and cellulose + polyacrylate material (P = 0.024) to *An. gambiae* s.l. Dispensing blend IB1 from polyester, cotton and cellulose + polyacrylate material had no influence on the responses of *An. gambiae* s.l. (P = 0.47) and *An. funestus* (P = 0.78). Furthermore, there was a lower response of *An. funestus* to IB1-impregnated nylon than to polyester (P = 0.022), cotton (P = 0.033) and cellulose + polyacrylate material (P = 0.012). Treatment had no effect on trap collections of *Culex* (P = 0.23), *Mansonia* (P = 0.79) and other anophelines (P = 0.45).

**Table 3:** **Mean number (±SE) of male mosquitoes caught in outdoor MM-X traps without odour bait, baited with blend IB1 dispensed from nylon, polyester, cotton and cellulose + polyacrylate material in Kigoche village for 25 nights**

Treatment	N	Mean number ± SE of mosquitoes caught				
		*An. gambiae*s.l.	*An. funestus*	*Culex*spp.	*Mansonia*spp.	Other anophelines
No odour bait	25	0.32 ± 0.11^a^	0.04 ± 0.04^a^	0.12 ± 0.07^a^	0.04 ± 0.04^a^	0.04 ± 0.04^a^
IBI-treated nylon	25	0.64 ± 0.16^a^	0.35 ± 0.12^a^	0.24 ± 0.09^a^	0.00	0.08 ± 0.06^a^
IB1-treated polyester	25	1.76 ± 0.27^b^	0.94 ± 0.20^b^	0.40 ± 0.13^a^	0.04 ± 0.04^a^	0.16 ± 0.08^a^
IBI-treated cotton	25	1.60 ± 0.25^b^	1.06 ± 0.21^b^	0.36 ± 0.12^a^	0.00	0.04 ± 0.04^a^
IB1-treated cellulose + polyacrylate	25	1.40 ± 0.24^b^	0.98 ± 0.20^b^	0.48 ± 0.14^a^	0.08 ± 0.06^a^	-

N is the number of experimental nights and SE is the standard error of the mean number of catches per night. Mean ± SE mosquito catches within the same column assigned different letter superscripts are significantly different at P < 0.05 (Generalized Linear Models).

### Abdominal status of major malaria vectors

**Figure 3:**
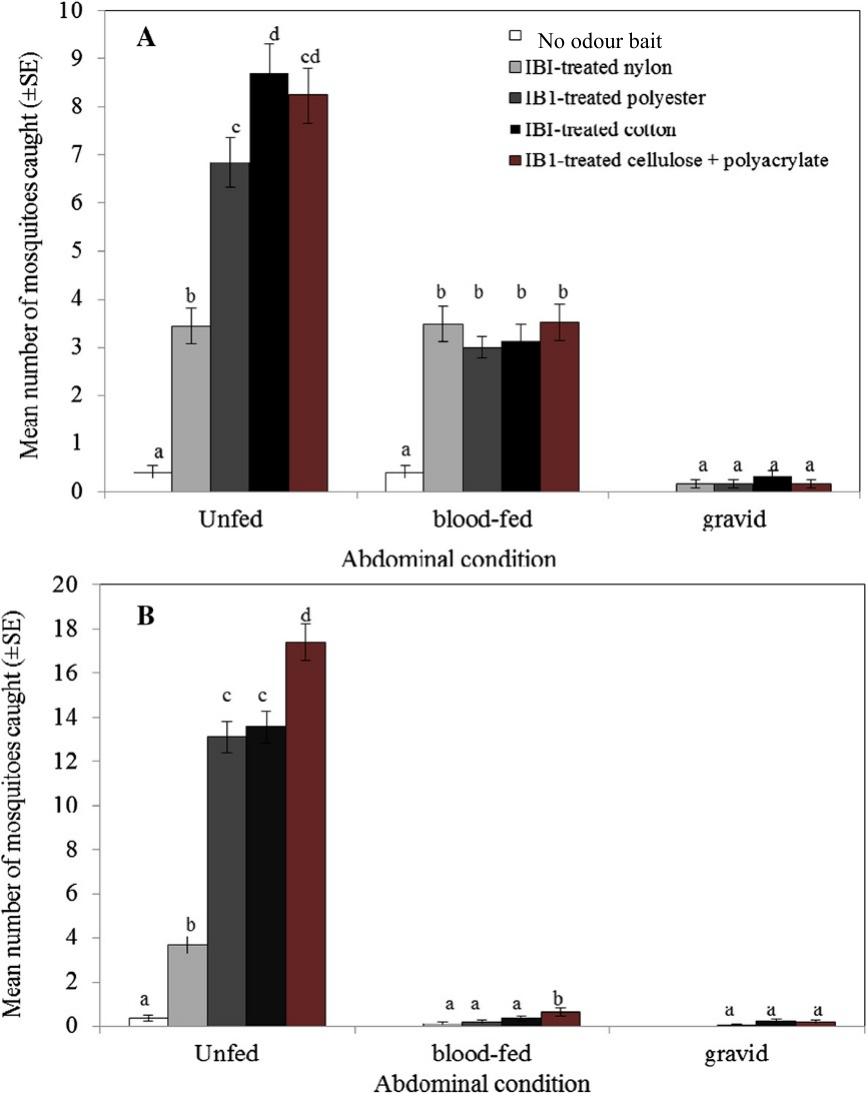
**Mean number ± SE of** ***An. gambiae*** **s.l. (panel A) and** ***An. funestus*** **(panel B) in different abdominal conditions (unfed, blood-fed and gravid) collected in an outdoor trap without odour (white square), baited with blend IB1 dispensed from nylon (gray square), polyester (dark gray square), cotton (black square) or cellulose + polyacrylate (brown square) material for 25 nights in Kigoche village.** Mean values with different letters within the same mosquito abdominal condition differ significantly (P < 0.05).

There were 1,049 female *An. gambiae* s.l. and 1,249 female *An. funestus* trapped. The majority of female *An. gambiae* s.l. collected were unfed (65.9%), whereas fewer were blood-fed (32.2%) and some were gravid (1.9%) (Figure 3A). Trap catches of unfed *An. gambiae* s.l. were significantly affected by treatment (P = 0.001). Dispensing of blend IB1 from nylon strips attracted a notably lower number of unfed *An. gambiae* s.l. compared to other substrates (P < 0.001). IB1-treated cotton and cellulose + polyacrylate material attracted the highest mean numbers of unfed *An. gambiae* s.l. that were similar for both materials (P = 0.74). Moreover, unfed *An. gambiae* s.l*.* responded equally to IB1-impregnated polyester and cellulose + polyacrylate material (P = 0.07). However, collections of blood-fed and gravid *An. gambiae* s.l. among the four IB1-impregnated materials were similar (P = 0.36 and P = 0.50, respectively).

Collected female *An. funestus* were largely unfed (96.4%), with few blood-fed (2.6%) or gravid (1%) mosquitoes (Figure 3B). The response of unfed *An. funestus* to IB1-baited traps was influenced by treatment (P = 0.001). Dispensing of blend IB1 from nylon caught significantly fewer unfed *An. funestus* compared to polyester (P = 0.001), cotton (P = 0.001) and cellulose + polyacrylate material (P = 0.001). Although there was no difference between the numbers of unfed *An. funestus* attracted to IB1-treated polyester and cotton materials (P = 0.67), each of these catches was significantly lower compared to IB1-impregnated cellulose + polyacrylate material (P < 0.001). Blood-fed *An. funestus* responded equally to blend IB1 dispensed from nylon, polyester and cotton (P = 0.43), however, cellulose + polyacrylate material was the most efficient substrate for dispensing of attractants (P < 0.041). Moreover, selection of dispensing material for blend IB1 had no impact on trap collections of gravid *An. funestus* (P = 0.25).

### Analysis of *An. gambiae*s.l. by PCR

Results from PCR analysis indicated that 117 out of 125 samples of *An. gambiae* s.l. were successfully identified translating into a success rate of 93.6%. All the 117 sub-samples were confirmed to be *An. arabiensis*. No *An. gambiae s.s* mosquitoes were found*.*

## Discussion

The release of Ifakara blend 1 from strips of cotton, polyester and cellulose + polyacrylate materials consistently lured more *An. gambiae* into traps compared to untreated nylon under semi-field conditions. Similarly, IB1-impregnated cotton, polyester and cellulose + polyacrylate materials attracted significantly more *An. gambiae* s.l., *An. funestus, Culex* and *Mansonia* species than IB1 dispensed from nylon strips under field conditions. Physiological status (unfed, blood-fed and gravid) differed among field-collected mosquitoes, the majority were unfed females of *An. gambiae* s.l. and *An. funestus. Anopheles arabiensis* was the only sibling species of the *An. gambiae* complex identified.

In all experiments, carbon dioxide was added to the synthetic blend to synergistically improve the attractiveness of synthetic odorants released from all four textile materials to mosquitoes [32–34]. Although it was recently established that nylon strips were more effective than LDPE sachets in dispensing synthetic mosquito attractants [13, 14], the present results demonstrate that alternative textile materials may perform equally well or even better than nylon for monitoring malaria mosquitoes. The better effect of polyester, cotton and cellulose + polyacrylate materials is possibly caused by a larger effective adsorbing capacity which allows for an even and constant dispensing of odorants to the environment. This seems to apply especially to the sanitary pads, consisting of cellulose + sodium polyacrylate. Cellulose provides fine fibres covered with sodium polyacrylate as a super adsorbent material. It is highly likely that a combination of the cellulose and the polyacrylate creates microfibers that are ideally suited for adsorption and slow-release of odorant compounds, thereby resulting in increased mosquito catches compared to nylon material.

The repeated utilization of the same IB1-impregnated substrates over 16 nights post-treatment under semi-field conditions followed by 25 consecutive nights of field testing confirmed their residual activity [14, 18]. This suggests that all substrates caused minimal change of the chemical properties of the impregnated active ingredients, leading to a sustained release of an attractive odour blend, thereby inducing a behavioural response over extended periods of time [11, 12].

Both cotton and polyester materials are preferable for disruption of the host-seeking process of endophilic malaria vectors as they can be impregnated with mosquito repellents and used as ceiling materials, window or door curtains [35, 36]. Repellent-impregnated cotton clothing could also be worn as an alternative solution against outdoor-malaria transmission or outbreaks of dengue transmitted by *Aedes aegypti* (L.), a vector species active during daytime [17, 37]. Polyester bed net material has also contributed substantially towards malaria reduction as such nets provide a long-term protection against mosquito bites and subsequent mosquito-borne diseases when impregnated with insecticides [15, 16, 38]. The textile materials were easy to use, locally available in different sizes and relatively cheap to be considered for large-scale application. Thus, the search for alternative and easy-to-prepare novel odour-dispensing systems can improve the effectiveness and sustainability of odour-baited technology as a tool for sampling, surveillance and control of host-seeking mosquitoes [2, 13, 36, 39]. Such systems should be evaluated for dispensing synthetic semiochemicals directed towards surveillance and disruption of mating, sugar-feeding and oviposition behaviour of mosquitoes [6, 40]. Nonetheless, these candidate attractant-treated matrices should be tested further for their wash-resistance and long-lasting residual activity on target mosquitoes as in the case of long-lasting insecticide-treated or repellent nets [16].

Females constituted 93.6% of all mosquitoes lured into outdoor traps baited with attractant-treated substrates compared to 6.4% males. The collection of significantly higher mean numbers of unfed female mosquitoes compared to blood-fed and gravid females irrespective of the type of odour-dispensing substrate proves that IB1 is a potent lure for sampling or control of female mosquitoes assumed to be host seeking [5, 7, 8]. The majority of the unfed mosquitoes caught are likely to have been newly emerged from adjacent irrigated rice fields, however, this was not determined during the study. Male mosquitoes do not require a blood meal instead they feed on plant nectar implying that the captured males are assumed to have been in pursuit of virgin females. It is also likely that a combination of synthetic odorants and volatiles produced by fermenting molasses mimics certain plant volatiles, which attracted male mosquitoes to the traps.

Whereas *An. arabiensis* is an opportunistic feeder, it was the only sibling species of the *An. gambiae* complex identified in our study area where existence of *An. gambiae s.s.* has been reported previously [10, 23]. *Anopheles gambiae s.s*. was the strain of choice for semi-field experiments but it was absent in outdoor mosquito collections possibly because of temporal and seasonal variation [41–43] as well as increased use of insecticide-treated bed nets [44]. The high catches of *An. gambiae* s.l. in a village where *An. arabiensis* is a primary malaria vector coupled with the fact that cows, goats and sheep were present adjacent to human dwellings, indicates that dispensing of blend IB1 from the tested materials served favourably as a human proxy [19, 45]. These results suggest that additional protection of people who are highly exposed to bites of *An. arabiensis* and *An. funestus* could be enhanced by deploying outdoor and indoor traps containing human-derived attractant-treated substrates and possibly by keeping insecticide-treated cattle to maximize the effects of zooprophylaxis [46–48].

Recent encouraging results have shown that a novel synthetic odour blend dispensed from nylon strips attracted as many *An. gambiae* s.l. but significantly more *An. funestus* compared to humans [10]. Similarly in Tanzania, a synthetic odour blend released from nylon attracted significantly higher numbers of *An. gambiae* s.l., *An. funestus, Culex* spp. and other anophelines than human volunteers when both were placed in separate huts [8]. Such findings demonstrate the prospects of deploying odour-baited technology for surveillance and disruption of indoor malaria transmission. With an intensified search for more potent synthetic attractants than humans and the addition of spatial repellents, a push-pull system could also be integrated into the prevention of both indoor and outdoor malaria transmission [49]. Targeting of outdoor-biting mosquitoes is currently important as recent studies have reported a shift from indoor- to outdoor-biting behaviour and transmission of malaria [50–52].

## Conclusion

The number and species of mosquitoes attracted to a synthetic odour blend is influenced by the type of odour-dispensing material used. Thus, surveillance and intervention programmes for malaria and other mosquito vectors using attractive odour baits should select an odour-release material that optimizes the odour blend. In such programmes, locally available cotton, polyester and cellulose + polyacrylate materials can effectively replace nylon.
